# Current Challenges and Future Directions in Handling Stroke Patients With Patent Foramen Ovale—A Brief Review

**DOI:** 10.3389/fneur.2022.855656

**Published:** 2022-04-28

**Authors:** Charlotte Huber, Rolf Wachter, Johann Pelz, Dominik Michalski

**Affiliations:** ^1^Department of Neurology, University of Leipzig, Leipzig, Germany; ^2^Department of Cardiology, University of Leipzig, Leipzig, Germany

**Keywords:** patent foramen ovale, PFO, cryptogenic stroke, PFO closure, secondary prevention, deep vein thrombosis, stroke

## Abstract

The role of patent foramen ovale (PFO) in stroke was debated for decades. Randomized clinical trials (RCTs) have shown fewer recurrent events after PFO closure in patients with cryptogenic stroke (CS). However, in clinical practice, treating stroke patients with coexisting PFO raises some questions. This brief review summarizes current knowledge and challenges in handling stroke patients with PFO and identifies issues for future research. The rationale for PFO closure was initially based on the concept of paradoxical embolism from deep vein thrombosis (DVT). However, RCTs did not consider such details, limiting their impact from a pathophysiological perspective. Only a few studies explored the coexistence of PFO and DVT in CS with varying results. Consequently, the PFO itself might play a role as a prothrombotic structure. Transesophageal echocardiography thus appears most appropriate for PFO detection, while a large shunt size or an associated atrial septum aneurysm qualify for a high-risk PFO. For drug-based treatment alone, studies did not find a definite superiority of oral anticoagulation over antiplatelet therapy. Remarkably, drug-based treatment in addition to PFO closure was not standardized in RCTs. The available literature rarely considers patients with transient ischemic attack (TIA), over 60 years of age, and competing etiologies like atrial fibrillation. In summary, RCTs suggest efficacy for closure of high-risk PFO only in a small subgroup of stroke patients. However, research is also needed to reevaluate the pathophysiological concept of PFO-related stroke and establish strategies for older and TIA patients and those with competing risk factors or low-risk PFO.

## Introduction

Clarifying individual stroke etiology is essential to establish the best possible secondary prevention. This is particularly important in light of a recurrence rate of 19.4% over 5 years (1). Based on the traditional “Trial of Org in Acute Stroke Treatment” (TOAST) classification, the causes large-artery atherosclerosis, cardioembolism, and small-vessel occlusion are distinguished from an undetermined stroke etiology, accounting for 20–30% of all infarctions (2). As undetermined stroke etiology also includes an incomplete evaluation and competing causes, the term cryptogenic stroke (CS) was introduced to characterize patients without risk factors despite adequate evaluation. This concept was further adapted by adding criteria like lesion size and localization, which resulted in a subgroup designated as “Embolic Stroke of Undetermined Source” (ESUS) (3).

Statistically, a patent foramen ovale (PFO) was found significantly more frequently in patients with CS and events classified as ESUS (4, 5). Nevertheless, the question arose whether the PFO is detected by chance since it occurs in about 25% of the general population (6), or whether there is a causal relationship, for example, in terms of paradoxical embolism (4, 7). The concept of paradoxical embolism includes a thrombus from the venous system [e.g., in the context of a deep vein thrombosis (DVT)] that enters the arterial system via a shunt between the atria, i.e., the PFO. From there, it can enter the brain, causing cerebral infarction (4). Cohnheim first described this theoretical principle in 1877 (8), followed by the first case reports in the 1980s with more or less direct evidence for an embolus that enters the PFO directly (9). However, the pathophysiologic concept underlying paradoxical embolism has not been fully elucidated. In detail, the relevance of DVT as a potential source for an embolus is still questionable since the PFO itself may also represent a prothrombotic structure.

Various studies compared antiplatelet therapy with oral anticoagulation as a drug-related approach to secondary prevention in stroke patients with coexisting PFO (10–13). Following the evidence emerging from pooled data, one meta-analysis found that anticoagulation is not superior to antiplatelet therapy (14), while another reported superiority of oral anticoagulation over antiplatelet therapy along with an increased risk of major bleeding (15). As the initial randomized trials failed to show superiority of PFO closure (16–18), individual benefit-risk assessments mostly led to antiplatelet therapy as the default therapeutic strategy in PFO patients with CS.

Better definition of high-risk PFO and patient profile led to proof of clinical benefit from PFO closure in three randomized trials (DEFENSE-PFO, REDUCE, and CLOSE), in terms of reduced secondary events after PFO closure compared to conservative therapy (13, 19, 20). Comparable observations were reported in the long-term course of a randomized trial that started several years earlier (21). However, in this study, a drop-out rate of 33.3% in the non-closure and 20.8% in the group with PFO closure occurred, particularly affecting the safety analyses because of unbalanced treatment groups (21). One crucial difference between the traditional and the latest randomized trials is, among others, that there were fewer specific inclusion criteria in the negative trials (16–18). In detail, the older studies also included those cerebral infarcts that were very unlikely to be due to a PFO. In contrast, the positive trials CLOSE (13) and DEFENSE-PFO (19) included only patients who had an atrial septal aneurysm or a pronounced right-to-left shunt in addition to the PFO. A further significant difference between the negative and positive trials is the included patient's age, while the trials showing efficacy for PFO closure only included younger patients (≤ 60 years). Another reason for the reported success might be technical developments in the last decade. Various percutaneous closure devices have been tested, while in 2018, two were approved by the US Food and Drug Administration: the Amplatzer PFO Occluder and the Gore Cardioform Septal Occluder (22). The choice of the device might also impact the rate of peri-procedural complications like new-onset atrial fibrillation. Even though a reliable differentiation between an already existing paroxysmal atrial fibrillation and a closure-related new-onset atrial fibrillation seems complicated, it was observed with a 4.59 times higher risk in a meta-analysis summarizing PFO closure studies, while the Amplatzer was a little superior to the Gore system (23).

At first glance, the latest randomized trials seem to provide robust evidence for handling a specific subgroup of stroke patients with coexisting PFO. However, a closer look reveals variations between the trials, especially regarding definitions of the qualifying cerebral event and the high-risk PFO, but also for the echocardiographic workup. Key features of the latest trials with details on study populations, inclusion criteria, and treatments are summarized in Table 1. The most varying issue here is the definition of a high-risk PFO, ranging from PFO with a right-to-left shunt (20) to detailed specification regarding septal hypermobility and PFO size (13, 19). Furthermore, one study restricted the echocardiographic workup concerning septal aneurysm to the group of PFO closure (20), preventing an accurate description of the overall study population.

**Table 1:** Comparisons of PFO closure vs. drug treatment alone in patients with stroke and PFO.

**Study**	**Patient's age**	**Qualifying cerebral event**	**Definition of high-risk PFO**	**Performed diagnostic workup**	**Device-related and drug treatment**	**Findings**
DEFENSE-PFO (19) PFO closure vs. drug treatment (either antiplatelet therapy or anticoagulation)	≤ 60 years, mean age 51.8 years	Ischemic stroke (clinical symptoms ≥ 24 h or radiological evidence) within previous 6 months, classified as cryptogenic stroke	PFO and septal aneurysm ≥ 15 mm, septal hypermobility ≥ 10 mm or PFO size ≥ 2 mm at rest or during Valsalva maneuver	TEE protocol including the use of agitated saline, performed prior to randomization	PFO closure group: closure plus dual inhibition of platelet aggregation at least for 6 months (up to local investigator) Drug treatment group: Single or dual inhibition of platelet aggregation or Warfarin (chosen by the local investigator)	Rate of stroke recurrence was lower in the PFO closure group than the group receiving drug treatment alone
REDUCE (20) PFO closure (with two versions of a closure device) plus antiplatelet therapy vs. antiplatelet therapy alone	18–59 years, mean age 45.2 years	Ischemic stroke (clinical symptoms ≥ 24 h or radiological evidence) within previous 6 months, classified as cryptogenic stroke	PFO and right-to-left shunt, classified by the number of microbubbles in the left atrium	TEE protocol that focused on the existence of PFO and right-to-left shunt including the use of agitated saline, performed prior to randomization Assessment of septal aneurysm was done at the time of PFO closure and thus only in the closure group.	PFO closure group: closure plus inhibition of platelet aggregation with at least clopidogrel for 3 days and then resume or start another (not specified) inhibition of platelet aggregation Drug treatment group: Aspirin alone, aspirin and dipyridamole, or aspirin and clopidogrel (up to local investigator)	Risk of ischemic stroke was lower in the PFO closure group than the group receiving antiplatelet treatment alone
CLOSE (13) PFO closure or anticoagulation vs. antiplatelet therapy alone	16–60 years, no information regarding mean age	Ischemic stroke (clinical symptoms and radiological evidence) within previous 6 months, classified as cryptogenic stroke	PFO and septal aneurysm ≥ 10 mm or large right-to-left shunt, defined as more than 30 microbubbles in the left atrium	TEE protocol (contrast agent not specified), performed prior to study inclusion	PFO closure group: closure and dual inhibition of platelet aggregation for 3 months, followed by single antiplatelet therapy Anticoagulation group: vitamin K antagonists or direct oral anticoagulants Group with inhibition of platelet aggregation: Aspirin, clopidogrel or aspirin combined with dipyridamol	Lower rate of stroke in the group with PFO closure plus long-term antiplatelet therapy than with antiplatelet therapy alone The effects of oral anticoagulation as compared with antiplatelet therapy on the risk of stroke recurrence could not be determined

In addition to these uncertainties, clinical practice frequently causes questions on the optimal therapy when considering the complexity of stroke etiology and the related diagnostic workup. Consequently, updated statements from neurological or stroke organizations mainly include PFO closure as an option only in patients with ESUS (24, 25), while one recommended PFO closure in CS (26). Reservations also become apparent by the differences in handling patients across European countries, even though PFO closure was shown as cost-effective, and volumes of procedures increased after the announcement of positive trial results in 2017 (27, 28).

This brief review summarizes current knowledge and challenges on handling stroke patients with coexisting PFO. Further, this work identifies open questions emerging in clinical practice, which might stimulate future research. Particular emphasis was given to the underlying pathophysiological concept of paradoxical embolism, different regimes of drug therapy independent of PFO closure, the special situation in patients older than 60 years with possible competing risk factors, and with transient ischemic attack (TIA).

## Methods

For this brief literature review, PubMed and Medline databases were used to identify studies about secondary prevention in patients with stroke and coexisting PFO or related pathophysiological aspects. The search included the period from the database's inception to October 2021. The following keywords were used: “patent foramen ovale,” “stroke,” and “closure,” “prevention” as well as the combination between “patent foramen ovale” and “DVT.” This search showed 698 results in total. Prospective studies, retrospective studies, *post-hoc* analyses from prospective studies and reviews were included. Records were excluded because of e.g., different article or study type, or because they did not fit thematically. The following themes were mainly focused: Pathophysiological aspects, morphological criteria of a PFO, drug- or device-related findings, and discussions on secondary prevention. There was no restriction by language. Generally, the applied methodology considered the concept of “Preferred Reporting Items for Systematic Reviews and Meta-Analyses” (PRISMA) (29).

## Results

### Pathophysiological Concept

The original rationale for PFO closure after stroke is based on the theoretical concept of paradoxical embolism. However, this concept does not seem to be the focus of the latest randomized trials (13, 19, 20), as factors like venous thrombosis were not part of the inclusion criteria. Considering the few available data that emerged from studies against the background of paradoxical embolism might help to understand the uncertainties on this topic.

The incidence of DVT or pelvic thrombosis in stroke is not studied regularly and mainly in a retrospective design. In one of the few available studies on the coincidence of DVT, a rate of 20% for deep leg vein or iliac vein thrombosis was found in young patients with CS compared to 4% in those with a determined etiology (30). Zietz et al. summarized findings from different studies with rates between 7 and 27% for DVT in patients with CS and PFO (31). However, Liberman et al. also examined the relationship between CS, PFO, and DVT (32). They found no significant difference in the rate of DVT between patients with PFO and CS as well as patients with PFO and a stroke of another cause. Moreover, one might assume that a thrombus originating from a DVT would be large enough to cause a large vessel occlusion in the brain. However, there is no data about the incidence of PFO in clinical trials that examined the endovascular treatment of ischemic stroke due to large vessel occlusion. As the currently available literature indicates a meager overlap rate between stroke patients with PFO and existing DVT, the PFO's causality is still a matter of debate, especially since the concept of paradoxical embolism is not yet fully understood.

In addition to the perspective of an embolism from the venous system, the PFO itself or an associated pathology is discussed to play a role as a thrombogenic structure. So far, there are not many studies describing local conditions of the PFO in so much detail that would allow conclusions about the thrombotic relevance of the PFO channel itself. Even though some case reports suggest the generation of a thrombus in a transit position [e.g., (33)], no systematic analysis of this presentation is available. However, the following characteristics qualify a high-risk PFO with increased risk of cerebrovascular events: Large shunt, larger PFO diameter during Valsalva, large septum excursion, and associated atrial septum aneurysm (ASA) (34, 35). Further morphological characteristics with an increased risk are: Tunneled PFO, coexisting right atrial septal pouch (RASP), hybrid defect, Eustachian ridge, significant shunt at rest, and PFO > 3 mm (36). Using data from DEFENSE-PFO and CLOSE, a *post hoc* comparison of patients who had a PFO with either large shunt or an associated ASA indicated that an ASA is a more important predictor of recurrent stroke than the shunt size (37).

For diagnostic workup, screening for DVT is not generally recommended in stroke patients with PFO because the probability of a positive finding and causality appears too low. Nevertheless, if there is clinical evidence of venous thrombosis, low-threshold diagnostics should be performed (31). Concerning echocardiography, the superiority of transesophageal (TEE) over transthoracic echocardiography (TTE) for detecting a PFO was reported in stroke patients (38, 39) and healthy subjects (6). TEE thereby allows a more accurate description of the PFO, i.e., the shunt size with an associated number of bubbles passing after applying a contrast agent (40). A further advantage of TEE is the more reliable detection of additional pathologies such as a septum aneurysm, which appears essential for classifying a high-risk PFO (38, 39). Consequently, TEE is recommended in patients with ESUS eligible for PFO closure (24, 35). Along with discussions on the echocardiographic workup, clinical practice with a likely higher frequency of TEE in younger stroke patients needs to be considered. Moreover, the rate of patients undergoing TEE may greatly vary even between specialist treatment centers (40). In addition to limitations originating from the applied echocardiographic technique, the fact of a potential false-negative examination, even when performed with contrast agent and by professionals, needs to be considered (6). In addition to echocardiography, transcranial Doppler (TCD) with bubble test is also seen as a potential technique to clarify the individual impact of the PFO (24). Thereby, TCD has a higher sensitivity but a lesser specificity than TEE (41), which is likely due to the lack of visualization of the PFO itself. Focusing on a potential genetic disposition for the existence of PFO, a recently published review failed to show a clear relationship to genetic variants, except for one that was seen in patients with atrial fibrillation (42). This finding might be of interest for future screening techniques and otherwise highlights the existing difficulties during diagnostic workup, especially the identification of patients with a not yet known paroxysmal atrial fibrillation.

### Anticoagulation and Inhibition of Platelet Aggregation in Patients With PFO

PFO closure was recently stated as a treatment option, not for all patients but younger ones with a high-risk PFO and CS in the absence of contraindications (24–26). If, for various reasons, a decision is made against closure, single or double platelet aggregation inhibition, or anticoagulation with Vitamin K antagonists (VKA) or novel (direct) anticoagulants (dOAC), or a combination of platelet aggregation inhibition and anticoagulation, seems possible. Many studies have examined the superiority of one over the other. At first, it appears that a tendency toward anticoagulation is recognizable, at least in highly selected patients. In detail, three randomized trials involving patients with ESUS or CS and coexisting PFO (NAVIGATE, PICCS, and CLOSE) described a slightly better outcome, in the sense of a lower rate of recurrent stroke, after anticoagulation (12, 13, 43). Comparable results were seen in a pooled analysis (15). However, considering a recent subgroup analysis of the RE-SPECT ESUS trial (14), the assumption of the advantage of anticoagulation in patients with ESUS and coexisting PFO needs to be reconsidered as a reduction of secondary events was not seen.

Discussing the potential usefulness of anticoagulation in stroke patients with PFO, conditions other than the PFO may trigger anticoagulation too. If a DVT is detected, a plasma coagulation inhibition is indicated anyway, at least for a while. Based on the theory of paradoxical embolism, in this situation, anticoagulation would, of course, represent the most suitable treatment to prevent embolic events, including stroke. On the other hand, if the PFO only coexists to stroke, secondary prevention with a platelet aggregation inhibitor might be more sufficient. Remarkably, the available studies often did not commit themselves to the applied drug concept. While studies comparing inhibition of platelet aggregation with anticoagulation focused on dabigatran or rivaroxaban (11, 14), the latest randomized trials that compared PFO closure with drug treatment alone did not describe precisely the type of anticoagulation or inhibition of platelet aggregation. Indeed, the site's investigator decided due to his own opinion, as specifications were not part of the study protocols (13, 19, 20). Although an individually chosen drug therapy is reasonable, it does leave an inaccuracy in the setting of a randomized trial. Drug-based secondary prevention consisted predominantly of VKA, dOAC (not specified), and dual or single inhibition of platelet aggregation. Mainly aspirin, clopidogrel, cilostazol, and extended-release dipyridamole were used. Mas and colleagues divided the patient collective into the subgroups PFO closure, anticoagulation (dOAC or VKA), and platelet aggregation inhibition (13). They observed a significant advantage of the occlusion compared to the inhibition of platelet aggregation. However, no conclusions could be drawn about the difference between anticoagulation and antiplatelet agents because the comparison was underpowered. Unfortunately, none of the latest three randomized trials included an in-depth protocol regarding the type and duration of drug-based therapy in addition to PFO closure (13, 19, 20). Often the concept of first double and then single antiplatelet treatment was chosen.

An example of the complexity of drug- and device-based secondary prevention in a real-life setting is a prospective case series published by Poli et al. (44). They stratified patients with CS and PFO into groups with PFO closure and drug treatment only. Forty-two of the 90 patients in the drug treatment only group had a low-grade PFO. Thirty-four (81%) of these received single antiplatelet therapy, and 8 (19%) received anticoagulation (7 dOAC, one VKA). Of the remaining 48 patients with high-risk PFO in the drug treatment only group, 19 (40%) received single antiplatelet therapy, and 29 (60%) received anticoagulation (23 dOAC, 6 VKA). The drug therapy range is clearly visible here, although this is a single-center study.

### Patients With Transient Ischemic Attack and Patients Older Than 60 Years

Patients with TIA are excluded from most studies. Possibly, because in the absence of radiological evidence for any infarction, it is even more challenging to determine an etiology. A separate TIA study or at least a subgroup analysis would be needed to make a statement about secondary prevention in this particular group of patients with a comparable risk for secondary events. Assuming the same pathophysiology in terms of paradoxical embolism or a prothrombotic PFO itself, the secondary prevention in patients suffering from TIA should not differ from that with a radiologically visible stroke lesion.

Another group not covered by the latest randomized trials is the patient collective over 60 years of age since only patients up to the age of 60 were included (13, 19, 20). After the recommendation of the closure of a high-risk PFO for those under 60 years of age, the question arises whether it is also advantageous for older patients or up to what age the patients benefit from closure. Essentially, risk factors that typically increase with age, such as the higher rate of atrial fibrillation and cancer (45, 46), must be considered as competing stroke causes. A randomized controlled study regarding this question has not yet been carried out, and large registries that would also help to explore the efficacy of PFO closure in the elderly stroke population with coexisting PFO are lacking. In a prospective case series, Poli and colleagues (44) treated patients with high-risk PFO and stroke classified as TOAST group 5b (undetermined etiology) differently depending on their age. Thereby, drug treatment alone (platelet inhibition or anticoagulation) was recommended for those over 70 years of age, and PFO closure for those under 70. They included patients with PFO and TIA or ischemic stroke between 2012 and 2016 (before the large positive randomized trials were published) and treated the patients according to local standards. Poli et al. (44) observed fewer recurrent strokes after closure than after drug treatment alone in the subgroup of those under 60 years of age. In contrast, there was no significant difference among those over 60. They also emphasized the low rate of recurrent stroke among the population with low-risk PFO, which is treated with medication alone, regardless of age.

## Discussion

Randomized trials suggest evidence for PFO closure in young patients with CS and high-risk PFO. However, as these trials did not consider venous thrombosis as an inclusion criterion and the echocardiographic workup regarding the PFO varied between the trials, a conclusion regarding the PFO's pathophysiological role cannot be derived from the trials. Indeed, the latest randomized trials indicated that in addition to the presence of a PFO *per se*, further factors, for instance, its morphological criteria, the patient's age, the individual risk factors, and finally, the assumed etiology of stroke, play an essential role in determining the indication for PFO closure. This concept seems to be supported by a recent meta-analysis, indicating the best effects in patients without concomitant risk factors, which was determined by two scores, i.e., the Risk of Paradoxical Embolism (RoPE) score and the PFO-Associated Stroke Causal Likelihood (PASCAL) score (47). However, this kind of evidence needs to be questioned as some uncertainties remain, for instance, regarding the most suitable technique for detecting a PFO. Furthermore, from a practical perspective, the definition for CS or even ESUS is relatively strict and requires a full diagnostic workup, including, for instance, prolonged cardiac monitoring and search for occult malignancy (48). This means that the frequency of diagnosing a CS or ESUS strongly depends on the effort made during the diagnostic workup. In this light, scores such as RoPE generally risk a false-positive rating toward a CS or ESUS in cases with an incomplete diagnostic workup. Moreover, in the latest randomized trials on PFO closure (13, 19, 20), the definition for the qualifying stroke varied and thus prevented a wide-ranging conclusion. Considering these facts, it can be deduced that the device-related treatment path in terms of a PFO closure may ultimately represent an option for a small proportion of stroke patients.

Taking up the traditional concept, there is the fundamental question of the actual paradoxical embolism rate in patients with stroke and coexisting PFO. Clarification would allow a reevaluation of the assumed pathophysiological concept of PFO-related stroke. Consequently, there is a need for studies addressing the coincidence of CS and DVT and the PFO itself as a potential prothrombotic structure.

Further, drug-based secondary prevention, both in addition to and as an alternative to PFO closure, but also in the situation of a coexisting DVT should be examined in more detail. Standardization of the concomitant drug treatment in the case of PFO closure would be favorable. However, this cannot be derived from the latest randomized trials because of high variations among the applied treatment regimes. Moreover, such an approach might be hampered by local standards and possible specifications arising from various devices. In a more general perspective, recommendations regarding the medical management after PFO closure, including, for instance, screening for thrombotic complications or necessary actions in case of hypersensitivities (49), would be advantageous. In the case of drug treatment alone, the aim should be to standardize the type and dose of platelet aggregation inhibition and to define a group of patients that might benefit from anticoagulation. This information might also help to choose the optimal drug-based therapy if a patient is against device-related interventions.

Focusing on the latest three randomized trials (13, 19, 20), especially the groups of patients over 60 years and low-risk PFO have not been adequately represented. Although the few available data indicated that older patients or local characteristics not reaching the criteria of a high-risk PFO would not benefit from PFO closure, a confirmation is necessary while using adequate study designs. One of the decisive factors might be the existence of atrial fibrillation, which increases sharply with age (45). In some cases, a clear differentiation between atrial fibrillation- and PFO-related stroke might certainly be impossible. A further group underrepresented in current research is patients suffering from a TIA. As the risk for subsequent stroke is also present in this group, it is essential to collect data on secondary prevention in TIA patients with coexisting PFO.

In addition to the still existing uncertainties regarding handling younger stroke patients and the optimal drug-based therapy, clinical practice regularly causes questions reflecting the overall stroke population. These questions focus on the optimal handling of stroke patients with coexisting PFO if they are older than 60 years, present a low-risk PFO or competing etiologies like atrial fibrillation, or suffer from a TIA. Further studies addressing single issues in a very concise way or large data sets covering multiple factors, such as morphological aspects of the PFO and other risk factors, are necessary to establish individual treatment strategies.

## Author Contributions

DM designed the study. CH performed the literature review. CH and DM wrote the manuscript. Critical revisions were made by RW and JP with an either cardiological or neurological focus. All authors contributed to the article and approved the submitted version.

## Conflict of Interest

The authors declare that the research was conducted in the absence of any commercial or financial relationships that could be construed as a potential conflict of interest.

## Publisher's Note

All claims expressed in this article are solely those of the authors and do not necessarily represent those of their affiliated organizations, or those of the publisher, the editors and the reviewers. Any product that may be evaluated in this article, or claim that may be made by its manufacturer, is not guaranteed or endorsed by the publisher.
